# Supplementary material for: Sequence element enrichment analysis to determine the genetic basis of bacterial phenotypes
Source: Nat Commun. 2016 Sep 16;7:12797. doi: 10.1038/ncomms12797 (PMC5028413; doi:10.1038/ncomms12797)
Supplement: Supplementary Information — Supplementary Figures 1-14, Supplementary Table 1 and Supplementary Methods. [file ncomms12797-s1.pdf]

Supplementary information

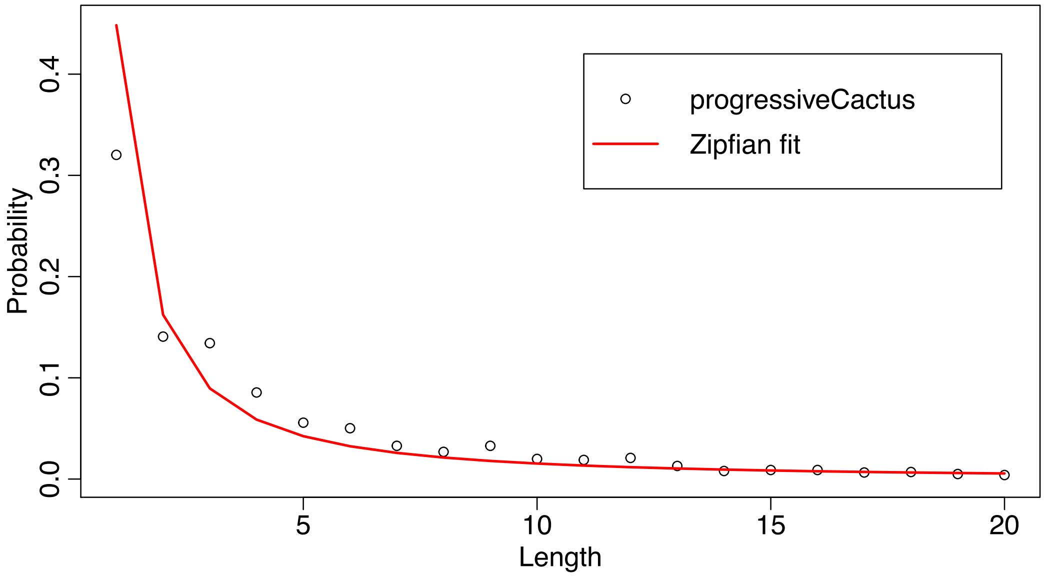

Supplementary figure 1: **Estimated size distribution for INDELs**
Estimated from a Progressive Cactus alignment of three members of the *Streptococcus* genus. A power law $p=L^k$ (Zipfian function; p is probability, L is INDEL length, k is a free parameter) is fit to the data. The observed distribution is used in the simulations.

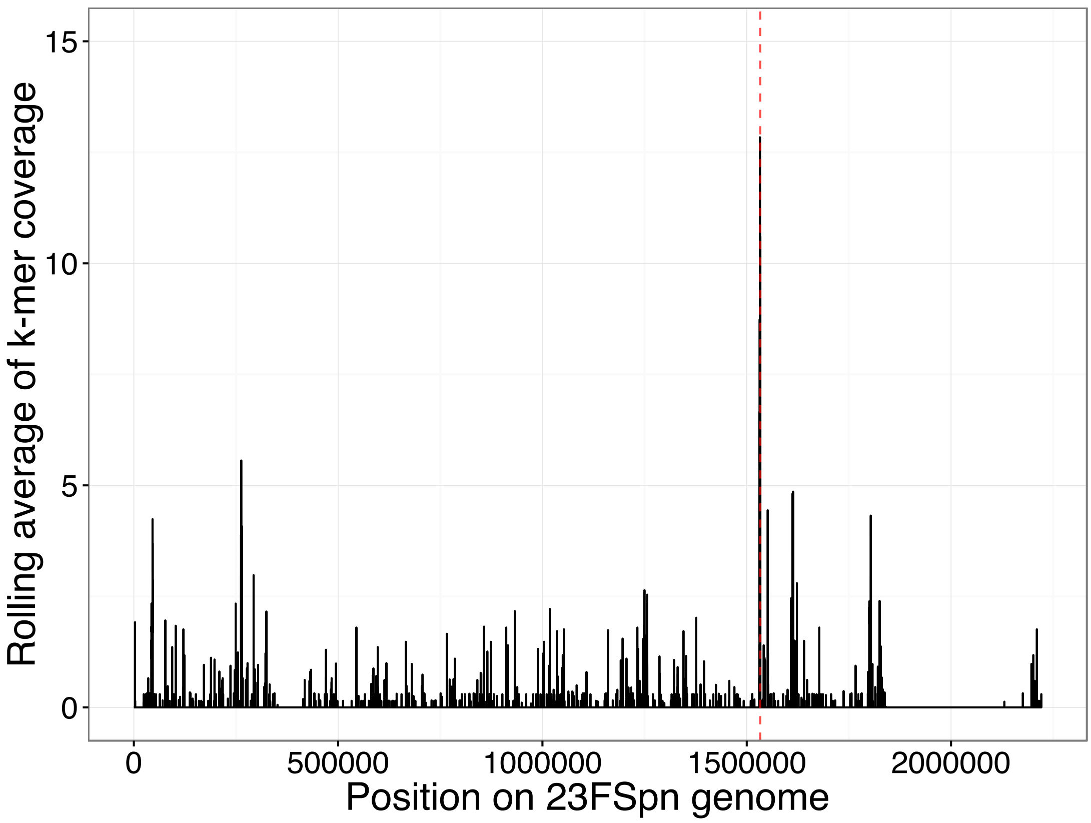

Supplementary figure 2: **Coverage of k-mers significantly associated with Trimethoprim resistance**.
K-mers are mapped to the 23FSpn reference genome. Plotted coverage is the rolling average over 100bp windows over the genome. The red dashed line at 1533003bp shows the location of the causal variant, overlapping with the peak in coverage.

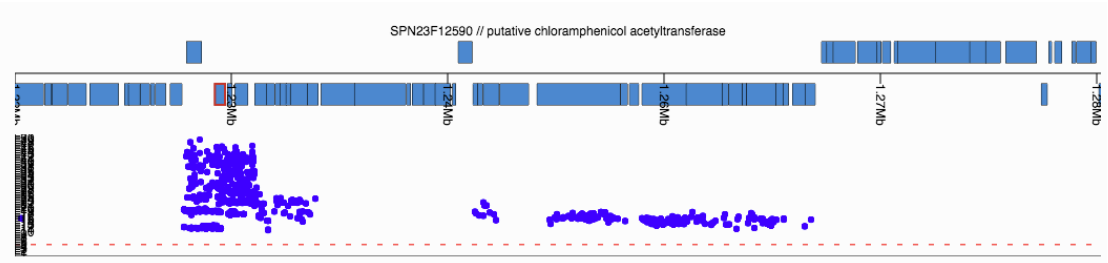

Supplementary figure 3: **Manhattan plot of chloramphenicol resistance** Phandango view of ATCC 700669 reference genome (blue blocks at top genes on forward and reverse strands) and Manhattan plot of start positions of the 1 508 of 1 526 k-mers significantly associated with chloramphenicol resistance which map to the integrative conjugative element (ICE) Tn*5253*. The hits are all within the ICE, and the most significant hits cluster around the *cat* gene (which is outlined in red; annotation appears at the top of the figure).

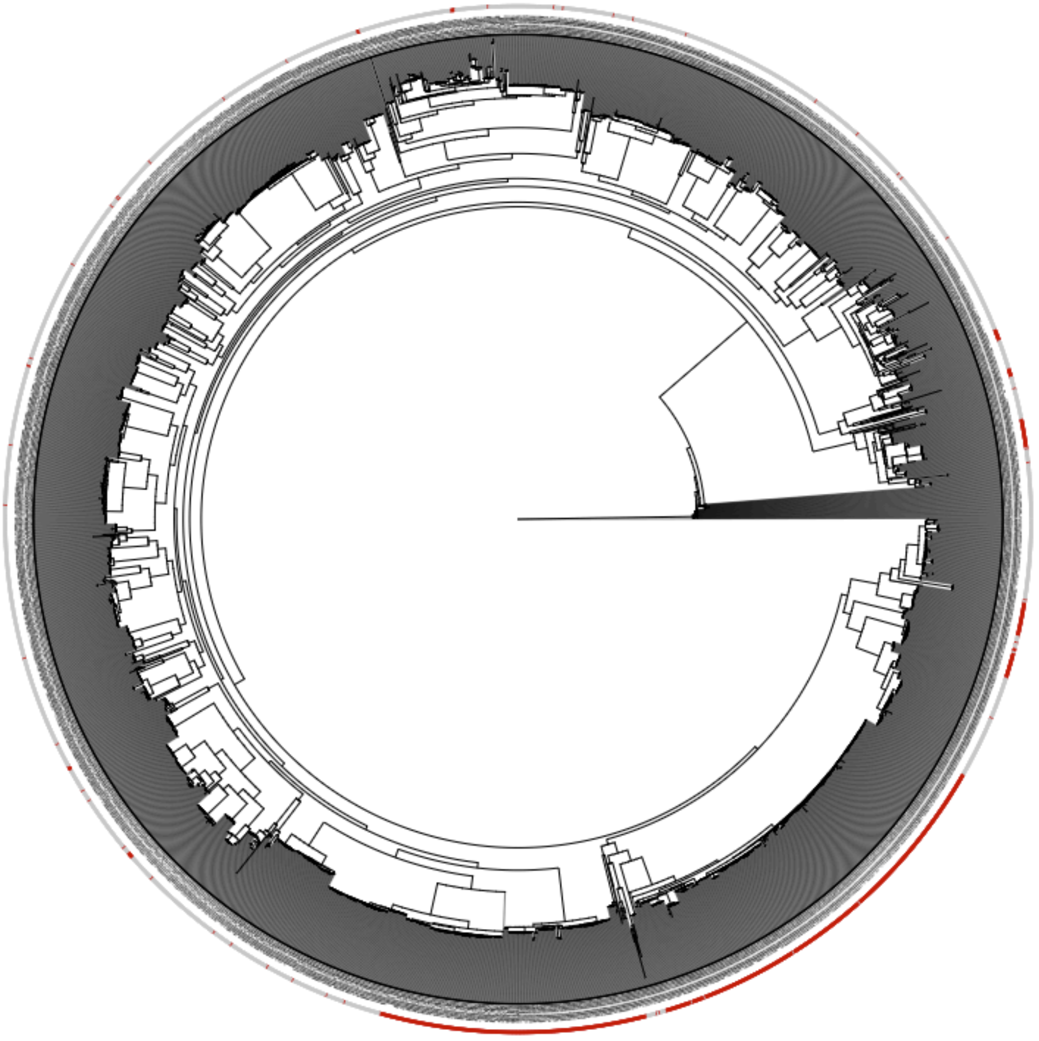

Supplementary figure 4: **Erythromycin resistance is clustered by lineage**
Neighbour joining tree from Chewapreecha *et. al.* study of 3069 Thai carriage *S. pneumoniae* samples based on SNP alignment produced by mapping to the ATCC 700669 reference strain. Outer ring: red if resistant to Erythromycin, grey if sensitive.

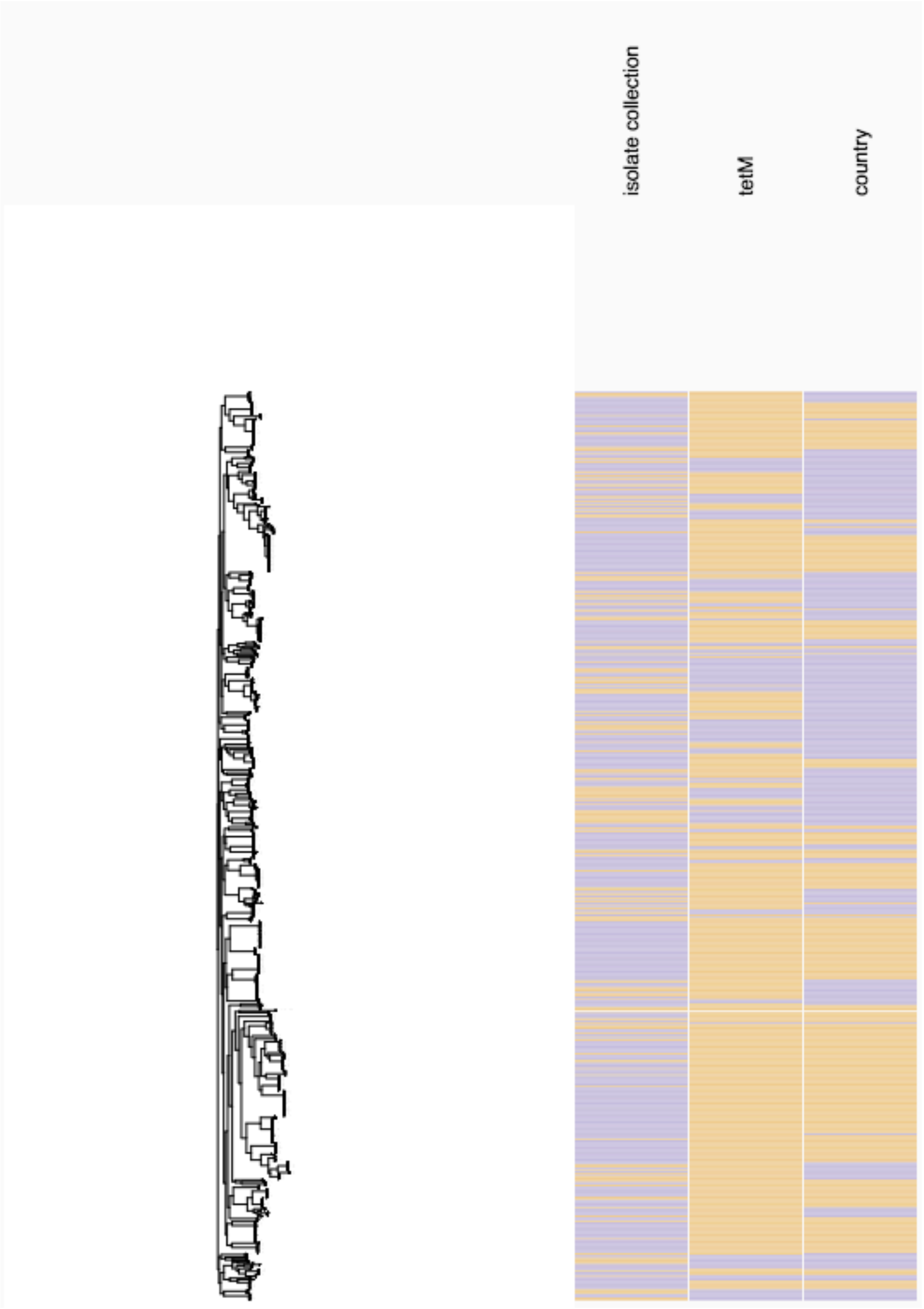

Supplementary figure 5: **Phylogeny and metadata for S. pyogenes data**
Phandango view of S. pyogenes metadata on the right, showing whether isolates are invasive/non-invasive (orange/purple), presence of *tetM* (orange – absent, purple – present) and country of isolation (orange – Fiji, purple – Kilifi). Tree from a core genome alignment of all isolates is drawn on the left, with tips aligned to the metadata.

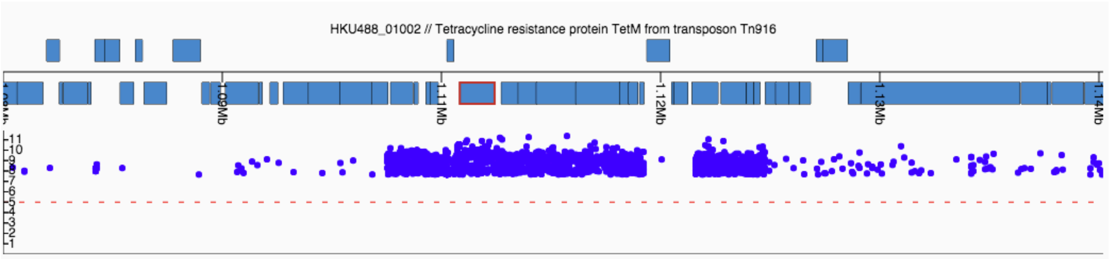

Supplementary figure 6: **Manhattan plot of Tn*916* associated with *S. pyogenes* invasiveness**

Phandango view of *S. pyogenes* HKU488 reference genome (blue blocks at top genes on forward and reverse strands, *tetM* highlighted in red) and Manhattan plot of start positions of k-mers significantly associated with invasiveness when not adjusted for country of origin.

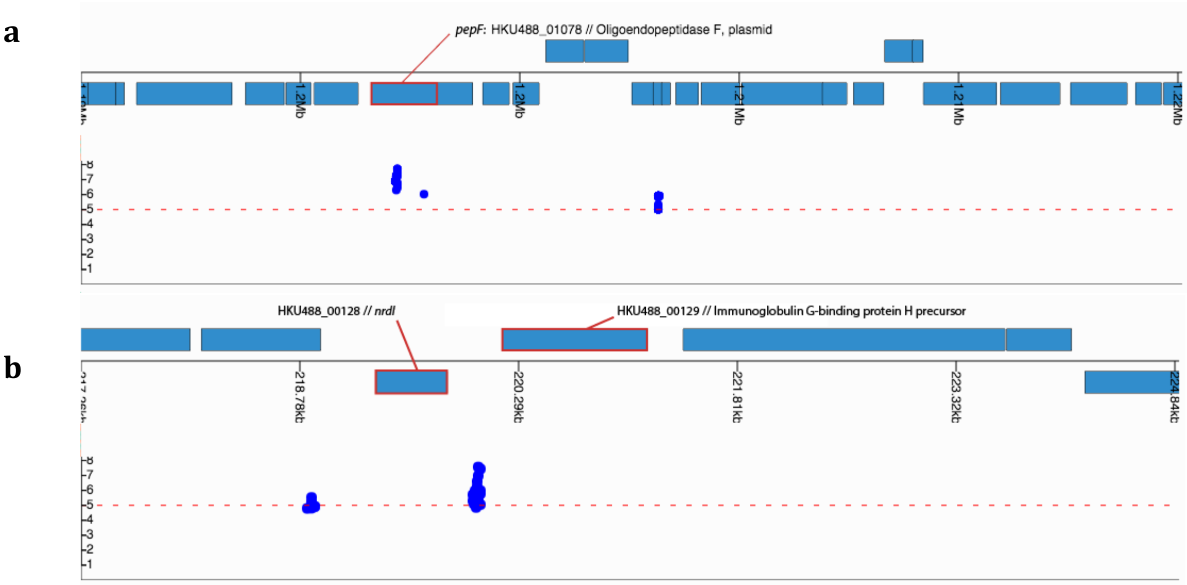

Supplementary figure 7: **Manhattan plot of loci associated with *S. pyogenes* invasiveness**

As supplementary figure 6, except with the Manhattan plot showing p-values when adjusted for country of isolation. The gene hit is surrounded by a red box, and the annotation shown at the top of the figure a) *pepF*; b) IgG-binding protein H precursor. Data are available, including full SEER output, at https://dx.doi.org/10.6084/m9.figshare.1613851.v2

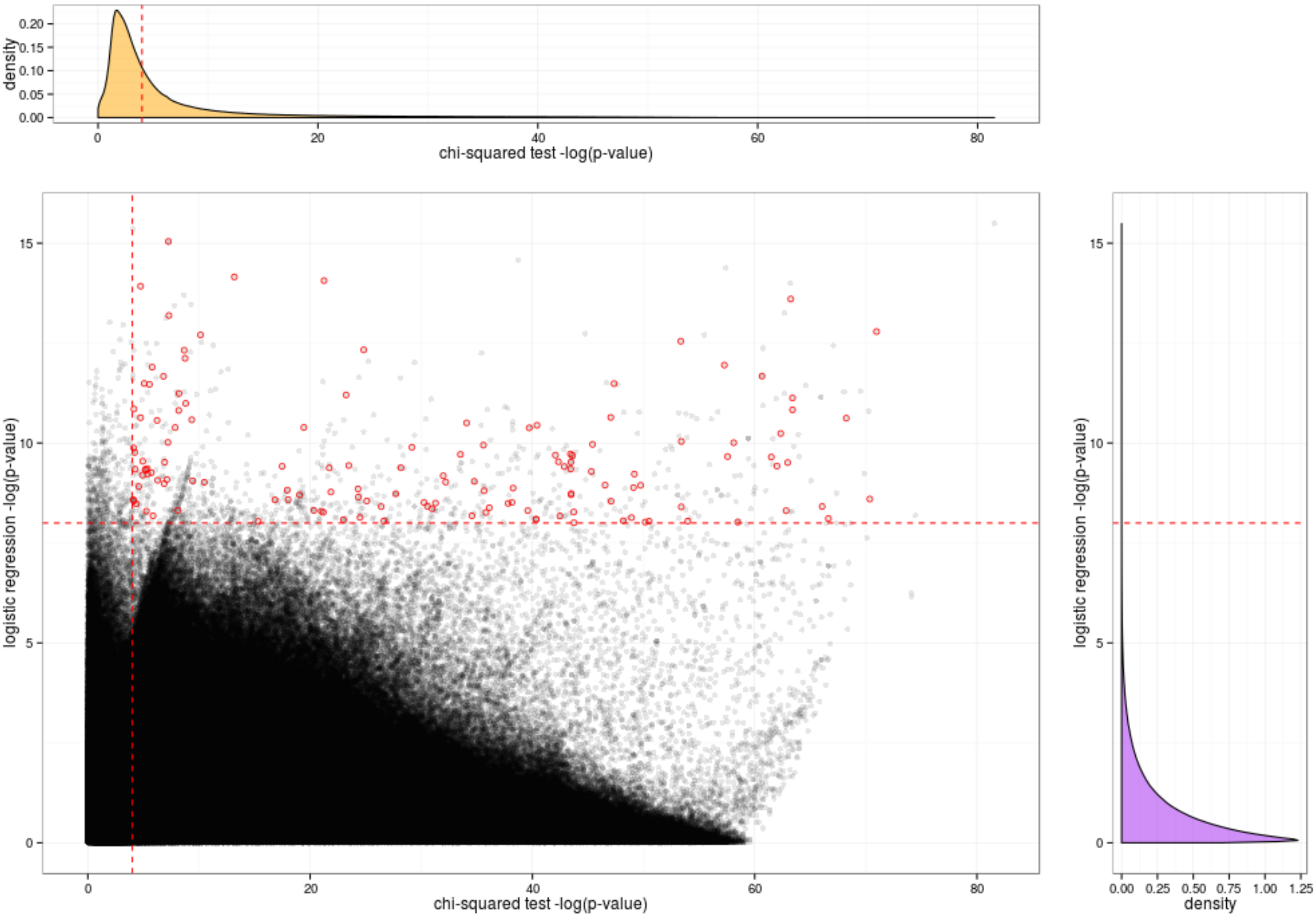

Supplementary figure 8: **p-p plot for simulated k-mers**

The $-\log_{10}$ p-values from a χ^2 test against the p-value from a logistic regression using the first three MDS components as covariates. The points are from all the simulated k-mers passing frequency filtering. k-mers meeting the threshold for significance (a cut-off of 1×10^{-8}) in the logistic regression) which map to the causal gene are coloured in red. The cut-offs used for each test are shown as red dashed lines. Top panel: marginal distribution of χ^2 p-values. Right panel: marginal distribution of logistic regression p-values.

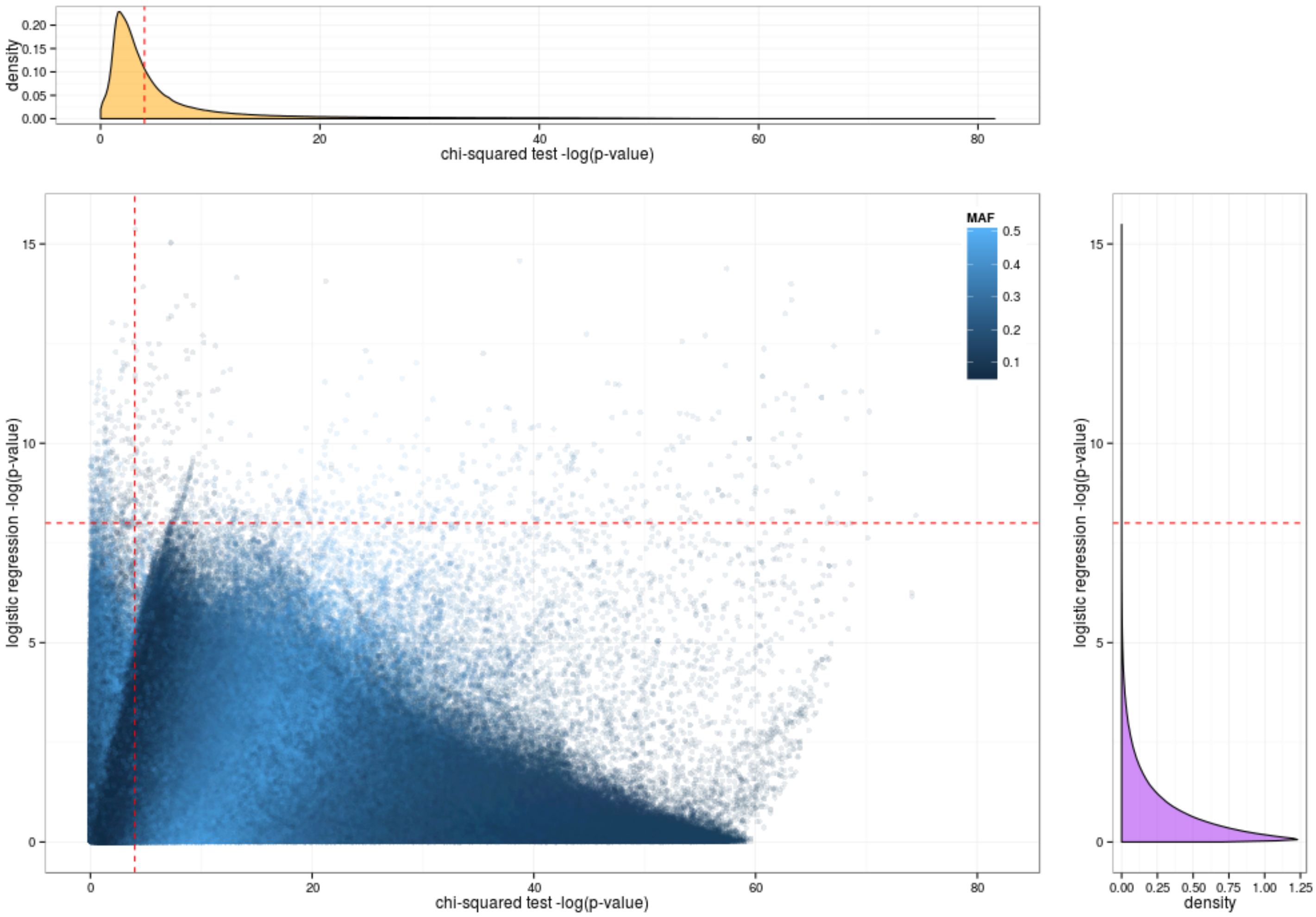

Supplementary figure 9: **p-p plot showing effect of k-mer frequency**
As supplementary figure 8, except the shading of each point (each representing one k-mer) is by minor allele frequency (MAF). Most of the k-mers with a high χ^2 p-value and low logistic regression p-value are at low frequency, as are those with equal p-values from each test.

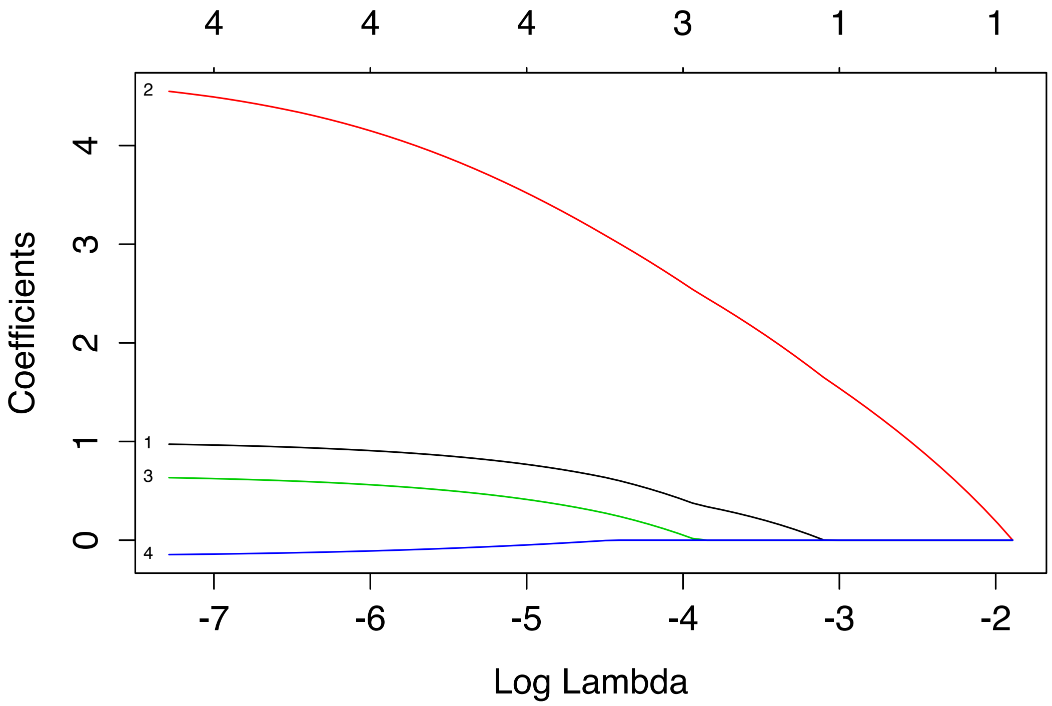

Supplementary figure 10: **Filtered k-mers are lineage driven associations**
Coefficients of a lasso logistic regression between phenotype and k-mer frequency vector plus the first three MDS components (for the k-mer with the lowest logistic regression p-value but a χ^2 p-value above the threshold for filtering – see top left corner of p-p plot in Supplementary figure 8). The x-axis is log of the l_1 regularisation parameter, with the left side of the graph representing the coefficients of a logistic regression with no regularisation. Labels along the top are the number of variables in the model. 1 (black) is the k-mer frequency vector, 2 (red) 1st MDS component, 3 (green) 2nd MDS component, 4 (blue) 3rd MDS component. The 1st MDS component enters the model before the k-mer frequency vector, and has a larger coefficient.

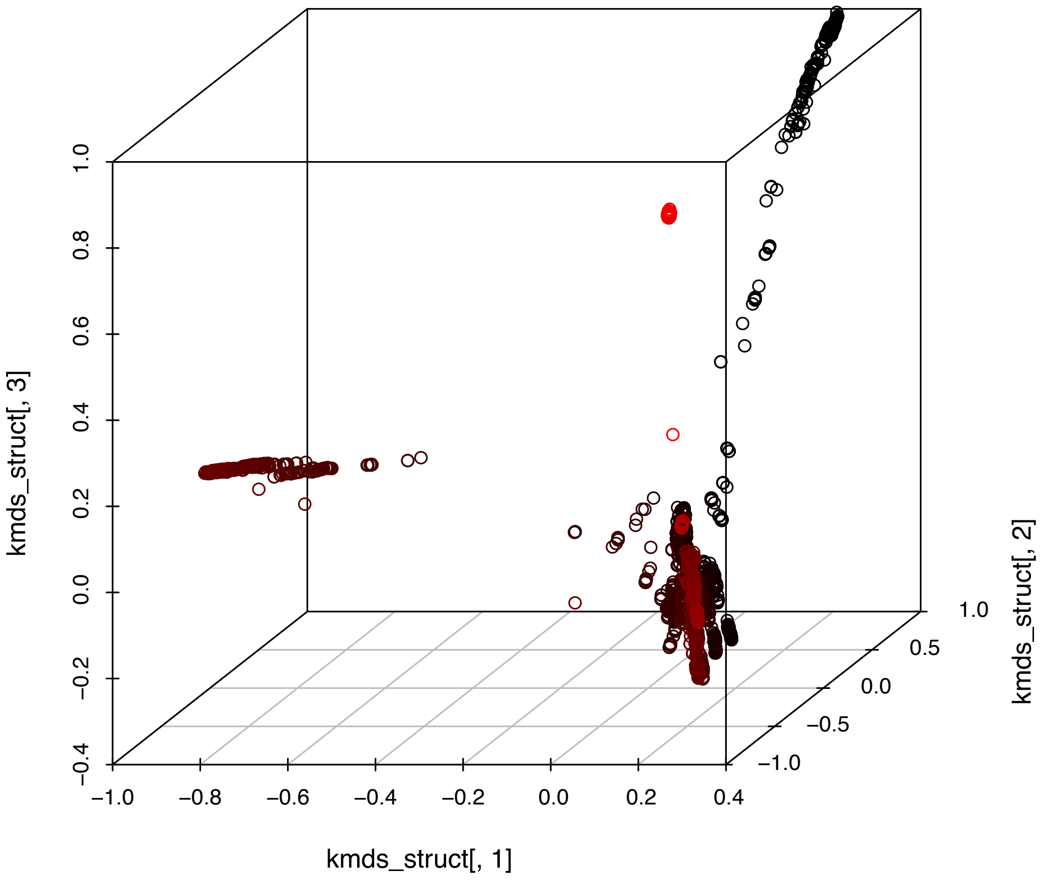

Supplementary figure 11: **k-mer distances projected into three dimensions** Projection of distance matrix **D** by MDS for the Chewapreecha *et. al.* study of 3069 Thai carriage *S. pneumoniae* samples. Shade from black to red is by y-coordinate (2nd MDS component).

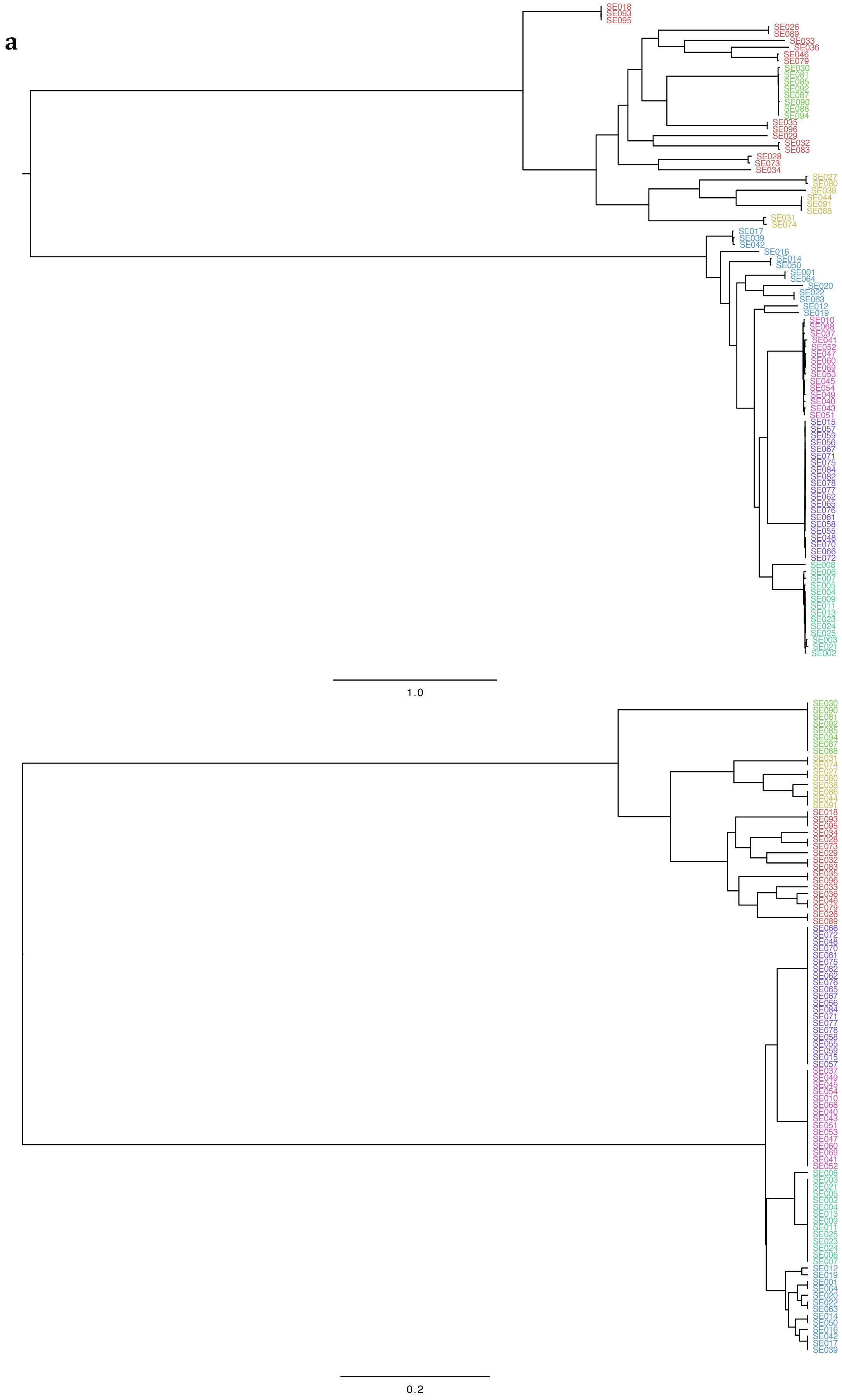

Supplementary figure 12: **Non-shared k-mers approximate population clusters**

Colours are hierBAPS clusters. a) Tree used for Monte Carlo simulations of 96 *S. pneumoniae* genomes. b) UPGMA tree from k-mer distance matrix produced from simulated reads.

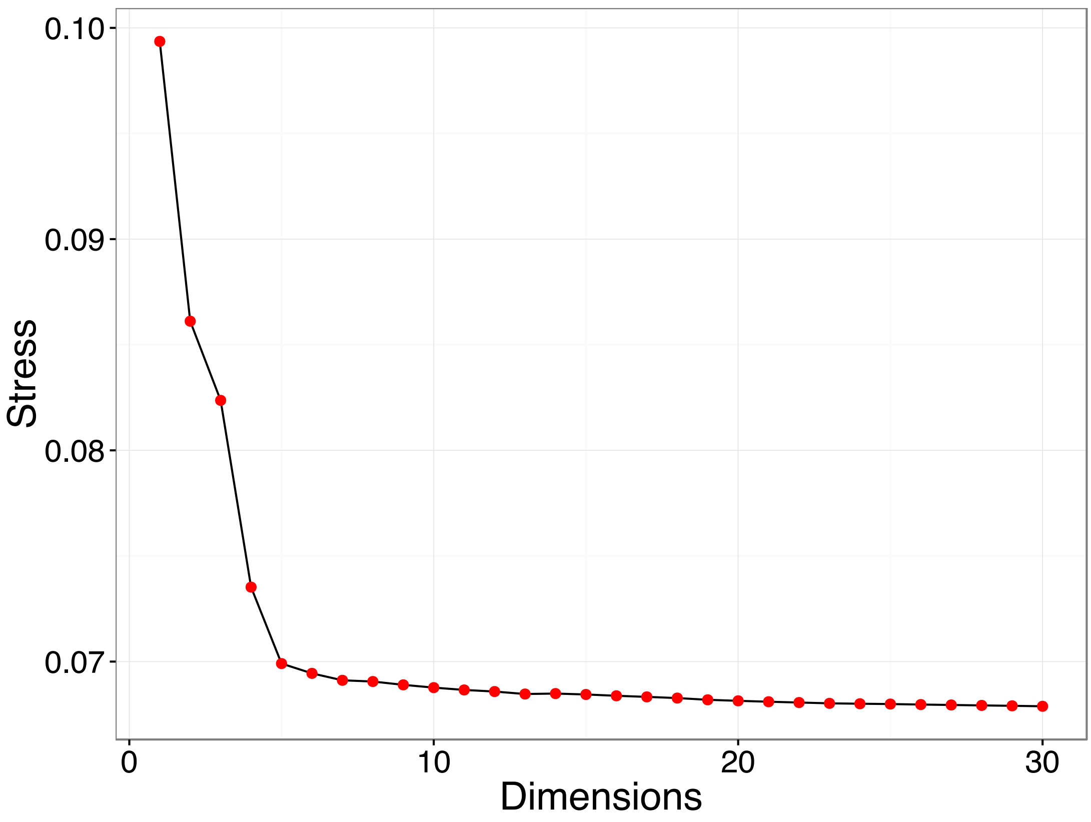

Supplementary figure 13: **Effect of number of dimensions used in MDS.** Stress against number of dimensions, calculated for the simulations in supplementary figure 12. Stress is defined as $S^2 = 1 - R^2$, where the R^2 statistic is calculated from a regression between the upper triangle of entries in the distance matrix (i.e. pairwise between all samples) and the Euclidean distance between samples in the reduced dimension space.

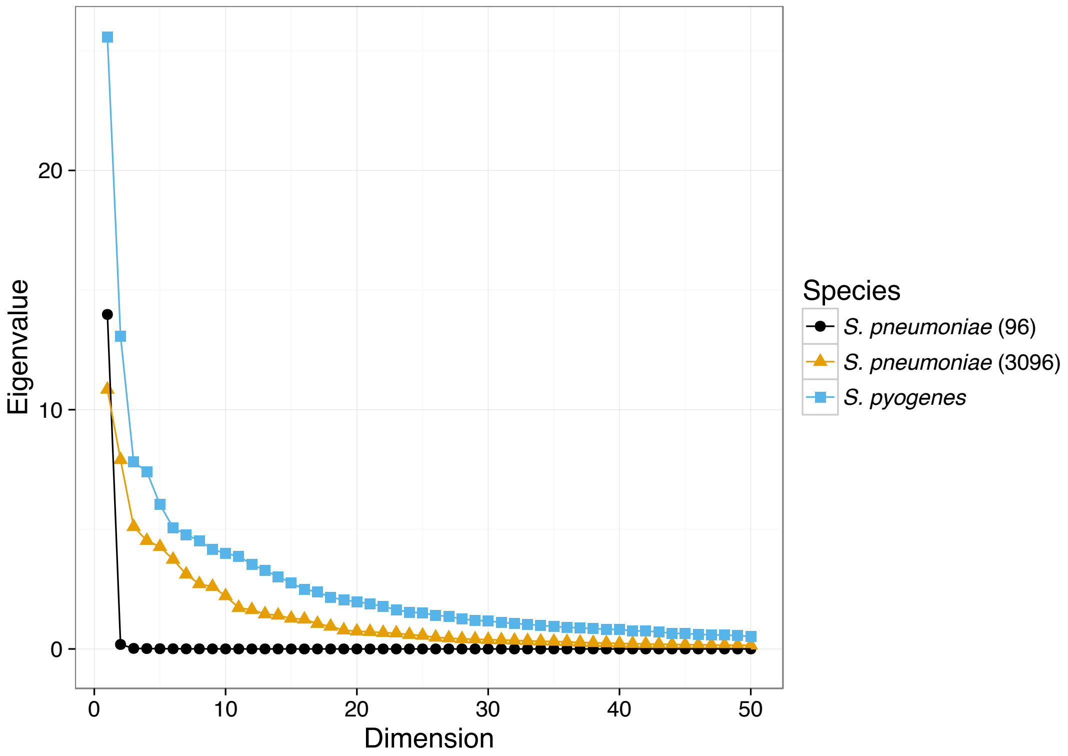

Supplementary figure 14: **Scree plot of MDS projections**
Eigenvalues for the first fifty dimensions of the 96 simulated *Streptococcus pneumoniae* isolates (Supplementary figure 12) in black, 3 069 *Streptococcus pneumoniae* isolates (Supplementary figure 4) in blue, and 675 *Streptococcus pyogenes* isolates (Supplementary figure 5) in orange.

Supplementary table 1: **Comparison of SEER with results from existing methods**

Antibiotic	Causal variant	Significant sites		Near correct site			Notes
		plink	dsk	plink			
Tetracycline	ICE, *tetM*	8 029	0	*tetM* – 124	ICE – 2240		
Chloramphenicol	ICE, *cat*	5 310	0	*cat* – 0	ICE – 1137		
β – lactams	*pbp2x*, *pbp1a*, *pbp2b*	858	0	*pbp2x* – 210	*pbp1a* – 113	*pbp2b* – 81	
Trimethoprim	*dyr* (I100L)	4 009	0	*dyr* – 47	*dpr* – 53		Causal SNP ranked 22nd
Erythromycin	*ermB*, *mef*, *mel*, *mefA*	8 469	0	None			Element not present in reference

The power to find genetic associations with antibiotic resistance in the Chewapreecha *et. al.* study of 3069 Thai carriage *S. pneumoniae* samples using existing methods. For each of the five antibiotics, the true causal variant is listed, as are the number of hits passing the significance threshold for each method (plink and dsk) and the number which map to the correct region.

Supplementary methods

Diverse simulated genomes

We used a gamma + invariant sites model as the distribution of rate heterogeneity among sites. As we didn't have estimates for the parameters of this distribution directly from our data, we used the estimate given by ALF. The resulting gamma distribution must have a longer tail than the real data, as some sites vary at high frequency. This creates many low-frequency k-mers.

As the simulation is computationally expensive to run, we reasoned that rather than running it lots of times with different parameters until a k-mer distribution identical to the observed data was reached we could use the original result as these low frequency k-mers would be filtered out in the common variation associations we are testing. 24.7M k-mers pass frequency filtering from the real data, whereas 12.7M pass from the simulated data – while this isn't quite the linear scaling expected with genome length (which would predict around 7M k-mers) the amount of common variation at the gene level is similar to real data.

For the purposes we use the simulations for, a gene driven association at different ORs, we believe this result is still an appropriate test. The genomes are related by a real phylogenetic tree with convergent evolution, gene loss and horizontal gene transfer – which are the key features being tested.
